# Molecular determinants of ATP-sensitive potassium channel MgATPase activity: diabetes risk variants and diazoxide sensitivity

**DOI:** 10.1042/BSR20150143

**Published:** 2015-08-18

**Authors:** Mohammad Fatehi, Chris R.J. Carter, Nermeen Youssef, Beth E. Hunter, Andrew Holt, Peter E. Light

**Affiliations:** * Department of Pharmacology, Alberta Diabetes Institute, Faculty of Medicine and Dentistry, University of Alberta, Edmonton, Alberta, Canada, T6G 2E1

**Keywords:** ATPase, diabetes risk variant, diazoxide, K_ATP_ channel

## Abstract

ATP-sensitive K^+^ (K_ATP_) channels play an important role in insulin secretion. K_ATP_ channels possess intrinsic MgATPase activity that is important in regulating channel activity in response to metabolic changes, although the precise structural determinants are not clearly understood. Furthermore, the sulfonylurea receptor 1 (SUR1) S1369A diabetes risk variant increases MgATPase activity, but the molecular mechanisms remain to be determined. Therefore, we hypothesized that residue–residue interactions between 1369 and 1372, predicted from *in silico* modelling, influence MgATPase activity, as well as sensitivity to the clinically used drug diazoxide that is known to increase MgATPase activity. We employed a point mutagenic approach with patch-clamp and direct biochemical assays to determine interaction between residues 1369 and 1372. Mutations in residues 1369 and 1372 predicted to decrease the residue interaction elicited a significant increase in MgATPase activity, whereas mutations predicted to possess similar residue interactions to wild-type (WT) channels elicited no alterations in MgATPase activity. In contrast, mutations that were predicted to increase residue interactions resulted in significant decreases in MgATPase activity. We also determined that a single S1369K substitution in SUR1 caused MgATPase activity and diazoxide pharmacological profiles to resemble those of channels containing the SUR2A subunit isoform. Our results provide evidence, at the single residue level, for a molecular mechanism that may underlie the association of the S1369A variant with type 2 diabetes. We also show a single amino acid difference can account for the markedly different diazoxide sensitivities between channels containing either the SUR1 or SUR2A subunit isoforms.

## Abstract (precis)

Molecular interactions between two residues in the sulfonylurea receptor (SUR) subunit of the ATP-sensitive potassium channel influence MgATPase activity. This interaction may provide a mechanism for the increased diabetes risk associated with a common channel variant and determines sensitivity to diazoxide.

## INTRODUCTION

ATP-sensitive potassium (K_ATP_) channels are key transducers linking cellular metabolic status to electrical excitability and therefore play important roles in many excitable tissues, including the endocrine, nervous, skeletal muscle and cardiovascular systems [1]. K_ATP_ channels are hetero-octameric transmembrane protein complexes comprised of four pore-forming inward rectifying potassium (K_ir_) 6× channel subunits coupled to four regulatory sulfonylurea receptor (SUR) subunits that possess intrinsic catalytic MgATPase activity [2,3]. Kir6.1 (*KCNJ8*), Kir6.2 (*KCNJ11*) and SUR1 (*ABCC8*) are encoded by individual genes, whereas SUR2A and SUR2B are splice variants of the *ABCC9* gene product. Differential subunit assembly results in distinct channel subtypes that exhibit cell/tissue-specific expression patterns, as well as different biophysical and pharmacological properties [4].

K_ATP_ channels function as molecular rheostats by adjusting cellular electrical excitability in response to alterations in cellular metabolism, primarily via their modulation by the intracellular nts ATP and ADP. The major site for the inhibitory action of ATP is located within the pore-forming Kir6.2 subunits [5]. In contrast, ADP can release the inhibition by ATP, leading to increases in channel activity via a complex molecular interaction within the SUR subunits. The SUR subunit contains two evolutionarily conserved hydrophilic nucleotide binding domains (NBDs) with each NBD containing a Walker A and B motif that bestow intrinsic MgATPase catalytic activity to the K_ATP_ channel subunit [6]. Adenine nucleotide-binding and ATP hydrolysis, generating ADP, take place in the catalytic region formed between Walker motifs. It is this MgATPase function that is considered important for regulating the appropriate K_ATP_ channel activity via ADP-induced K_ATP_ channel activation [7,8]. This is highlighted by the fact that human mutations in SUR1 that increase MgATPase activity underlie several cases of rare monogenic neonatal diabetes [9]. Furthermore, we have previously shown that the common diabetes susceptibility variant S1369A (rs757110) in SUR1 results in increased MgATPase activity and channel activation that may contribute to the development of type 2 diabetes by suppressing insulin secretion [2]. We speculated that the removal of the side chain hydroxy group in the S1369A variant results in a loss of a hydrogen bond with the side chain of the Gln^1372^ residue in the hairpin loop region adjacent to the catalytic MgATPase site in NBD2 [2]. However, this putative mechanism remains to be conclusively determined at the molecular level.

Diazoxide is commonly used to treat rare monogenic hyperinsulinaemia resulting from certain inactivation mutations in either the *ABCC8* or *KCNJ11* genes, although the precise molecular mechanism by which diazoxide acts is unknown. K_ATP_ channels containing the SUR1 subunit are activated by the K_ATP_ channel opener diazoxide; whereas the channels containing the SUR2A subunit possesses a substantially different pharmacology [10], requiring ADP to be present [11,12]. Interestingly, diazoxide is thought to increase K_ATP_ channel activity via increasing the intrinsic MgATPase activity of the channel complex [9,13,14]. Therefore, diazoxide sensitivity may involve regions important for regulating MgATPase activity, such as the residues in the hairpin loop adjacent to the major MgATPase catalytic site in NBD2.

## EXPERIMENTAL

### Cell culture, transfection and electrophysiology

tsA201 cells, a HEK293 cell line derivative, were cultured in Dulbecco's modified Eagle's medium (DMEM)/FBS and then transfected with the Kir6.2 and SUR1 clones using the calcium phosphate precipitation technique [15]. Transfected cells were identified using fluorescent optics in combination with co-expression of a GFP plasmid (Life Technologies). Macroscopic K_ATP_ channel recordings were then performed 48–72 h after transfection. The excised inside-out patch clamp technique was used to measure macroscopic recombinant K_ATP_ channel currents in transfected tsA201 cells as described in detail previously [15]. Experiments were performed at room temperature (21°C). The bath solution for recording in the absence of Mg^2+^ ions contained 2 mmol/l EGTA and no MgCl_2_. Beryllium fluoride (BeF_2_) was dissolved in 50 mmol/l KF [33% (w/v)] to produce a sufficient amount of the ATP γ-phosphate mimetic BeF_x_ (BeF^3−^ and BeF_2_^4−^) [16]. In bath solutions used for experiments in the presence of BeF_x_, 50 mmol/l KCl was replaced with 50 mmol/l KF.

### Experimental compounds

BeF_2_, MgATP, diazoxide and Na^+^ salts of GTP and GDP were obtained from Sigma–Aldrich. ATP, GTP and GDP were prepared as 10 mmol/l stock solutions in double-distilled water (ddH_2_O) immediately prior to use.

### Site-directed mutagenesis

The full-length human SUR1 and SUR2A DNA constructs were a generous gift from Dr J. Bryan (Pacific Northwest Diabetes Research Institute, Seattle, WA). All mutants used in the present study were generated using site-directed mutagenesis (QuickChange, Stratagene) and subsequently confirmed by sequence analysis.

### *In silico* homology modelling

The homology model of the SUR1 NBD1 and NBD2 dimer [17], based on the prokaryotic ATP-binding cassette (ABC) protein crystal structure of MJ0796 (PDB accession # 1F30) was generously provided by Dr C.G. Nichols (Washington University, St. Louis, MO) and was used to visualize and predict the location and interactions of key regions and residues in the NBD1/2 dimer using Pymol software [17]. These computer models were used to represent hypothetical residue interactions for the purpose of illustration.

### Cysteine cross-linking

Disulfide bond formation was induced by exposing excised patches expressing wild-type (WT), single and double cysteine mutant SUR1 subunits to 0.3% H_2_O_2_ for 100 s. The effect of H_2_O_2_ oxidation on WT and mutant receptors was assayed by measuring the amplitudes of currents elicited by 1 mmol/l MgGTP in the presence of 0.1 mmol/l MgATP before and after H_2_O_2_ application. The reversibility of disulfide bond formation was examined by exposing patches to the reducing agent DTT (10 mmol/l for 100 s) and measuring the current amplitudes induced by 1 mmol/l MgGTP in the presence of 0.1 mmol/l MgATP.

### Biochemical MgATPase assays

Four 681-bp long fragments of NBD2 (encoding amino acids 1301–1528), containing either WT SUR1 (Ser^1369^), WT SUR2A (Lys^1337^) or the mutants SUR1 (Lys^1369^) and SUR2A (Ser^1337^) were sub-cloned into pGEX-4T-1 GST fusion protein expression vectors. The recombinant plasmids were sequenced and then transformed into BL21 (DE3) cells for protein expression. The GST–NBD2 fusion proteins were detected with a monoclonal anti-GST-tag antibody (1:5000 dilution, Santa Cruz Biotechnology). Following purification, proteins (50 μg) were incubated at 40°C for 30 min followed by 1 h incubation at 4°C to allow homogeneous dimerization of NBD2 monomers. All experiments were performed in an ATPase activity buffer at 37°C using ADP-free ATP (ATP-Gold DiscoverX) as a substrate. MgATPase activities of NBD2 dimers were determined by monitoring ADP formation via coupling to production of the fluorescent product resorufin (ADP Quest, DiscoverX). Resorufin formation was monitored continuously (*λ*_ex_=560 nm, *λ*_em_=591 nm) in black 96-well plates in a SPECTRAmax Gemini XPS microplate spectrofluorometer (Molecular Devices). Initial rates of resorufin formation were plotted compared with ATP concentration and data were fitted to a rectangular hyperbola with the Michaelis–Menten equation (GraphPad Prism 6.0f) to obtain *V*_max_ and *K*_M_ values. All values are given as mean ± S.E.M. Significance was determined by use of an unpaired two-tailed *t*test.

### Statistical analysis

Macroscopic K_ATP_ channel currents were normalized and expressed as changes in test current relative to control current. Macroscopic current analysis was performed using pClamp 10.0 (Axon Instruments) and Origin 6.0 software. Statistical significance was assessed using the unpaired Student's *t*test or one-way ANOVA with a Bonferroni post hoc test. *P*<0.05 was considered statistically significant. Data are expressed as the mean ± S.E.M.

## RESULTS

### Predicted amino acid side chain interactions and their effects on MgATPase activity

Previous work in our laboratory has shown that the side chain of the Ser^1369^ residue may form a hydrogen bond with Gln^1372^ and that the common genetic S1369A variant is predicted to demonstrate a loss of this interaction. This leads to increased MgATPase activity and K_ATP_ channel activation in pancreatic β-cells that may contribute to the observed increase in susceptibility to type 2 diabetes associated with this variant [2]. These two residues are on opposite sides of a hairpin loop in the β-sheet forming the backbone of the NBD2 portion of the major catalytic MgATPase site 2 in SUR1 (Figure 1A). This initial work led us to speculate that the relative strength of the molecular interaction between the side chains at positions 1369 and 1372 may have an effect on the dynamic flexibility of the adjacent β-sheets and therefore influence MgATPase activity in a predictive manner. Therefore, we hypothesized that an increase in the 1369–1372 side chain interaction strength would decrease flexibility and result in reduced MgATPase activity, whereas a decrease in the 1369–1372 side chain interaction strength would result in increased flexibility and an increase in MgATPase activity. In order to explore the effect of side chain interactions between residues 1369 and 1372, we used previously published predictions of adjacent β-sheet side chain interactions [18] to generate a panel of mutations at positions 1369 and 1372 in SUR1 (Table 1). We decided to generate mutations in these residues that we predicted would either: 1) decrease side chain interactions and increase MgATPase activity (Ala^1369^, Ala^1372^ and Leu^1372^); 2) have no effect on side chain interactions or MgATPase activity (Gln^1369^, Ser^1372^, Thr^1369^ and Gln^1369^/Ser^1372^); or 3) increase side chain interactions and decrease MgATPase activity (Asn^1372^, Thr^1369^/Asn^1372^ and Lys^1369^; Table 1; Figure 1). WT or mutant SUR1 subunits were co-expressed with WT Kir6.2 subunits and the inside-out patch technique was used to measure macroscopic K_ATP_ channel currents in response to application of GTP that acts as a substrate for MgATPase activity, generating GDP that stimulates K_ATP_ channel activity. Therefore, an increased GTP response to GTP is a convenient way to measure MgATPase activity by electrophysiology as, unlike ATP, GTP does not possess an inhibitory effect on channel function [2,14]. Based on predicted interaction values in Table 1, substitution of either residue with an alanine (Ala^1369^ or Ala^1372^) should result in a weaker interaction and a larger GTP response, indicative of increased MgATPase activity. As predicted, removal of the side chains with the Ala^1369^ and Ala^1372^ mutations each resulted in a significant increase in GTP-activated current when normalized to WT GTP response: 163±14% and 132±4% respectively (*P*<0.01, Figures 1B–1D and 1F). To reduce interactions while maintaining side chain length, we generated the Leu^1372^ mutation (Figure 1E). As predicted, this mutation resulted in a significant increase in GTP-induced current (111±4%) compared with WT (*P*<0.01; Figures 1E and 1F).

**Table 1:** Estimation of amino acid side chain interactions Predicted strengths of amino acid side chain interactions. Values are based on amino acid pairing preferences in parallel β-sheets of proteins by Fooks et al. [18]. 1 Å=0.1 nm.

Position 1369 (sidechain radii Ǻ)	Position 1372 (side chain radii Ǻ)	Mutation(s) tested	Interaction value (change relative to WT)	Predicted change in I_MgGTP_ (relative to WT)
(S) Serine (0.71)	(Q) Glutamine (1.86)	WT	1.5 (None)	(None)
(A) Alanine (0.00)	(Q) Glutamine (1.86)	Ala^1369^(risk)	0.9 (decrease)	Increase
(S) Serine (0.71)	(A) Alanine (0.00)	Ala^1372^	0.8 (decrease)	Increase
(S) Serine (0.71)	(L) Leucine (1.64)	Leu^1372^	0.8 (decrease)	Increase
(Q) Glutamine (1.86)	(Q) Glutamine (1.86)	Gln^1369^	1.4 (no change)	None
(S) Serine (0.71)	(S) Serine (0.71)	Ser^1372^	1.5 (no change)	None
(T) Threonine (1.33)	(Q) Glutamine (1.86)	Thr^1369^	1.5 (no change)	None
(Q) Glutamine (1.86)	(S) Serine (0.71)	Gln^1369^/Ser^1372^	1.5 (no change)	None
(S) Serine (0.71)	(N) Asparagine (1.59)	Asn^1372^	1.9 (increase)	Decrease
(T) Threonine (1.33)	(N) Asparagine (1.59)	Thr^1369^/Asn^1372^	3.0 (increase)	Decrease
(K) Lysine (2.27)	(Q) Glutamine (1.86)	Lys^1369^	2.2 (increase)	Decrease

**Figure 1:**
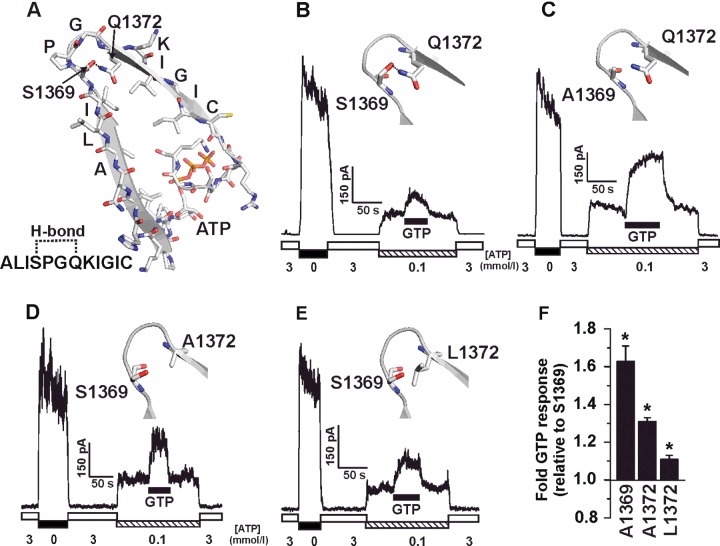
Amino acid substitutions resulting in a significant increase in GTP-induced K_ATP_ channel current (**A**) *In silico* model of the hairpin loop in the β-sheet region proximal to the MgATPase activity site 2 in NBD2. The hydroxy group on the side chain of residue Ser^1369^ is predicted to form a hydrogen bond with the terminal NH_2_ group of the side chain on the glutamine at position 1372 (Gln^1372^). Dotted line denotes the hydrogen bond. (**B–E**) Stimulation of K_ATP_ channels via the application of 0.1 mmol/l ATP and 1 mmol/l GTP in the WT (Ser^1369^/Gln^1372^) and Ala^1369^, Ala1372 and Leu^1372^ mutant SUR1 K_ATP_ channels respectively. (**F**) Grouped data of normalized GTP-stimulated K_ATP_ channel current. *n*=10–17 patches per group, *P<0.01.

We then tested the effects of mutations that are predicted to possess similar side chain interactions to WT (Ser^1369^ and Gln^1372^) and not significantly alter the observed GTP response (Table 1). The single mutations Gln^1369^, Ser^1372^ and Thr^1369^ did not result in a significant change in GTP-induced K_ATP_ channel activity: 107±4%, 107±6% and 102±3% respectively when compared with WT (*P* > 0.05; Figures 2A–2C and 2E). The reversal of residues from Ser^1369^/Gln^1372^ in WT SUR1 in the Gln^1369^/Ser^1372^ double mutant did not produce any change in the GTP response: 101±9% (*P* > 0.1; Figures 2D and 2E).

**Figure 2:**
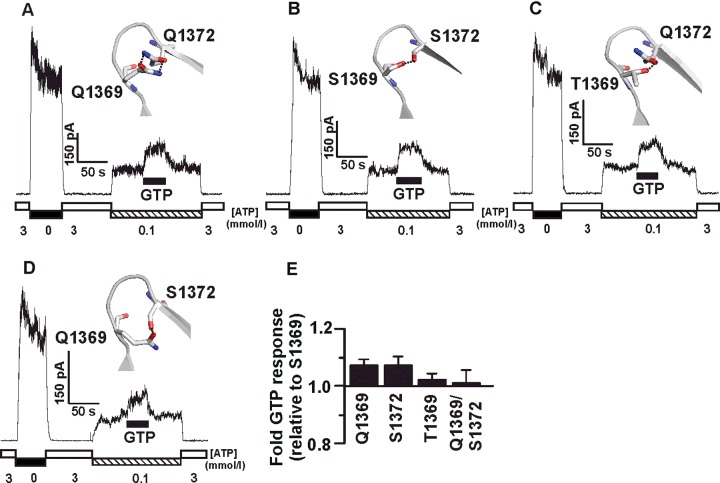
Amino acid substitutions that do not significantly alter GTP-induced K_ATP_ channel current (**A**–**D**) Stimulation of K_ATP_ channels via the application of 0.1 mmol/l ATP and 1mmol/l GTP in the Gln^1369^/Gln^1372^, Ser^1369^/Ser^1372^, Thr^1369^ and Gln^1369^/Ser^1372^ mutant SUR1 K_ATP_ channels respectively. (**E**) Grouped data of normalized GTP-stimulated K_ATP_ channel current. (*n*=5–7 patches per group).

Finally, we investigated the Asn^1372^, Thr^1369^/Asn^1372^ and Lys^1369^ mutations that are predicted to increase the strength of the interaction between residues 1369 and 1372, resulting in a decreased GTP response. All three mutants, Asn^1372^, Thr^1369^/Asn^1372^ and Lys^1369^, demonstrated a significant reduction in GTP-induced channel activity: 91±3%, 89±5% and 69±3% respectively when compared with the WT response (*P*<0.01; Figures 3A–3D).

**Figure 3:**
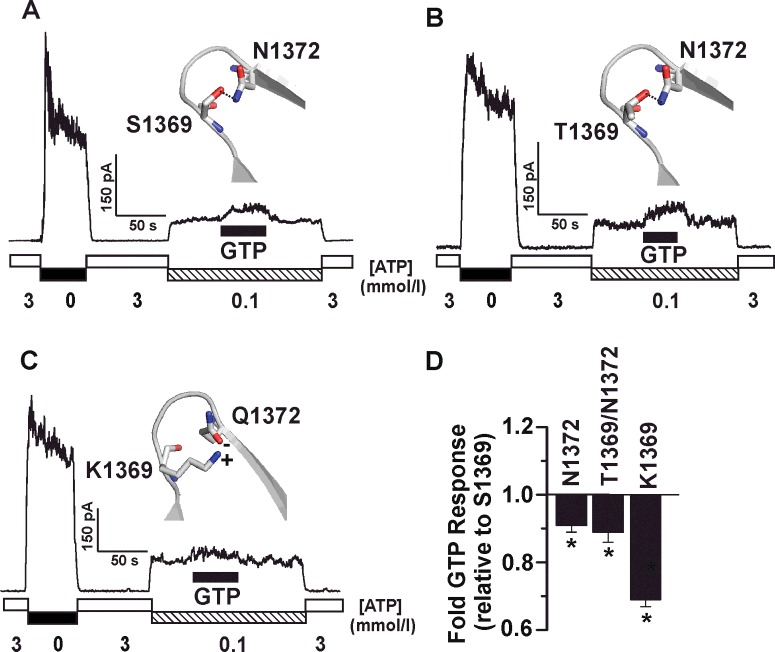
Amino acid substitutions resulting in a significant decrease in GTP-induced K_ATP_ channel current (**A**–**C**) Stimulation of K_ATP_ channels via the application of 0.1 mmol/l ATP and 1mmol/l GTP in the Asn^1369^, Asn^1369^/Thr^1372^ and Lys^1369^ mutant SUR1 K_ATP_ channels respectively. (**D**) Grouped data of normalized GTP-stimulated K_ATP_ channel current. (*n*=6–14 patches per group). *P<0.01.

### Disulfide trapping

To characterize further the side chain interactions taking place between residues in position 1369 and 1372, we employed a cysteine mutagenic strategy to evaluate the effects of reversible covalent bond formation between these two residues by generating a single SUR1 Cys^1369^ mutant (as control) and the double Cys^1369^/Cys^1372^ mutant pairing in SUR1 (Figure 4). We utilized H_2_O_2_ (0.3%, 100 s) as the oxidizing agent to promote disulfide bond formation between Cys^1369^ and Cys^1372^, with the disulfide-bond reducing agent DTT (10 mmol/l, 100 s) being used to break the disulfide bond. Application of H_2_O_2_ did not result in a significant change in GTP response in either WT SUR1 (Ser^1369^ and Gln^1372^) or the single cysteine Cys^1369^ mutant SUR1 (Figures 4A–4B and 4D). However, when compared with the WT GTP response, the GTP response in the double cysteine mutant Cys^1369^/Cys^1372^ was significantly reduced from 176±5% before H_2_O_2_ exposure to 130±5% after H_2_O_2_ exposure (*P*<0.01, Figures 4C and 4D). Application of DTT returned the GTP response to values similar to those obtained prior to H_2_O_2_ exposure (167±3%; Figures 4C and 4D).

**Figure 4:**
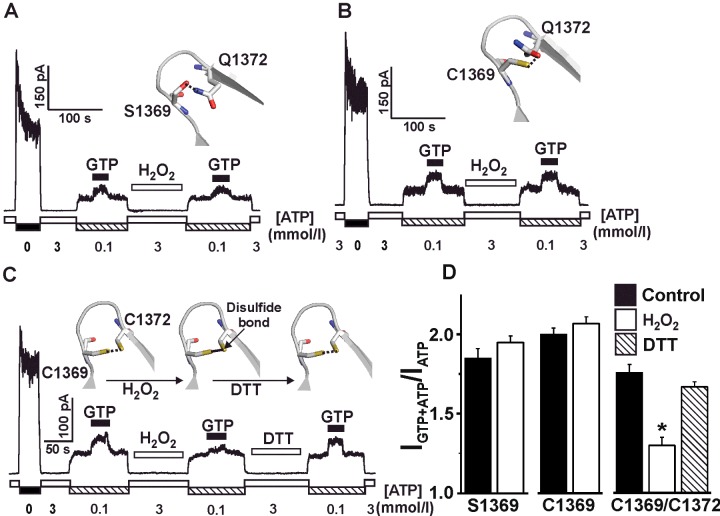
Disulfide bond formation decreases GTP-induced K_ATP_ channel function in a reversible manner (**A** and **B**) Stimulation of K_ATP_ channels via the application of 0.1 mmol/l ATP and 1mmol/l GTP in the WT Ser^1369^/Gln^1372^ and Cys^1369^ single cysteine mutant SUR1 K_ATP_ channels respectively. Exposure to 0.3% H_2_O_2_ did not result in a significant change in current magnitude. (**C**) Exposure to 0.3% H_2_O_2_ significantly reduces GTP-stimulated current of the double cysteine SUR1 mutant Cys^1369^/Cys^1372^. Current is restored to previous value following application of 10 mmol/l DTT and disulfide bond reduction. (**D**) Grouped data of normalized GTP-stimulated K_ATP_ channel current (*n*=4–9 patches per group). *P<0.01

### Biochemical MgATPase activity from NBD2 dimers

We decided to use a direct assay of MgATPase activity in several selected GST–NBD2 protein dimers. Of particular interest is the large reduction in GTP response observed in a SUR1 Lys^1369^ mutant when compared with WT (Ser^1369^). Furthermore, sequence alignment of the NBD2 catalytic region reveals that the only residue difference between SUR1 and SUR2A NBD2 is the presence of a lysine (Lys^1337)^ in SUR2A at the homologous residue to Ser^1369^ in SUR1 (Figure 5A). SUR1 and SUR2A containing channels also possess different properties, including MgATPase activity [19,20]. Therefore, we used a biochemical enzymatic assay to measure directly the MgATPase activity in NBD2 dimers from WT SUR1 (Ser^1369^) and WT SUR2A (Lys^1337^) respectively, as well as from the reverse mutants SUR1 (Lys^1369^) and SUR2A (Ser^1337^) by generating the corresponding GST–NBD2 dimers. Since the relative concentrations of catalytically-active NBD2 dimers present in our assays could not be determined, kinetic data from the MgATPase assays could not provide useful information regarding effects of introducing serine or lysine on relative catalytic rate constants and thus on measured *V*_max_ values. However, the *K*_M_ values are significantly lower in NBD2 dimers containing a lysine residue such as the WT SUR2A Lys^1337^ (38±6 μmol/l) and mutant SUR1 Lys^1369^ (30±4 μmol/l) compared with the NBD2 dimers with a serine residue in the same positions (WT SUR1 Ser^1369^, 135±6 μmol/l and the SUR2A Ser^1337^ mutant, 160±12 μmol/l); the shifts in *K*_M_ values are most clearly evident in plots where V values are expressed as a fraction of the (arbitrary) measured *V*_max_ values (Figure 5B).

**Figure 5:**
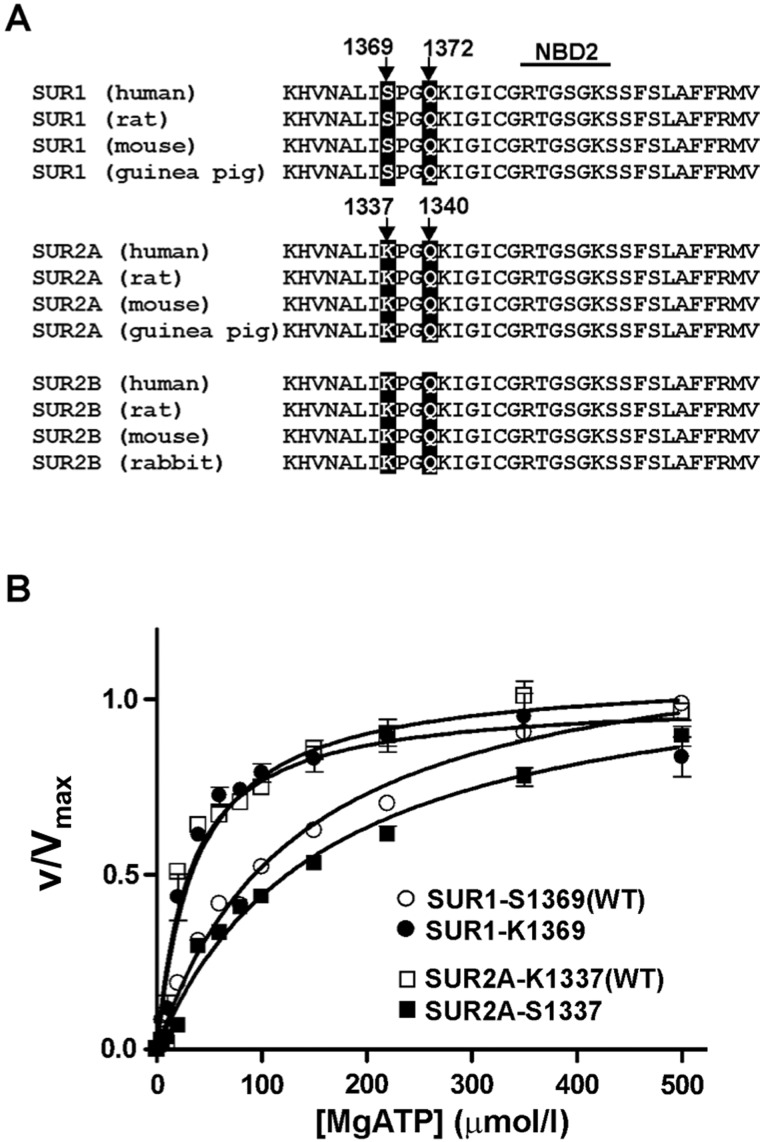
Direct MgATPase assay of NBD2 dimers (**A**) Amino acid sequence alignments of the SUR1 and SUR2A NBD2 domains. Homologous residues SUR1 Ser^1369^ and SUR2A Lys^1337^ are highlighted. (**B**) Hyperbolic plot of normalized MgATPase values. *n*=3–5 per group.

### The effect of a positively charged lysine residue at position 1369/1337 on diazoxide sensitivity and GTP-induced K_ATP_ channel activity

Previous work has suggested that the ability of diazoxide to evoke currents from the K_ATP_ channel is dependent on MgATPase activity [11,13] and the SUR2A subunit is not activated by diazoxide unless ADP is also present [21]. Therefore, we investigated the role of the hairpin loop Ser^1369^ residue in SUR1 and its homologue, Lys^1337^ in SUR2A (Figure 5A) on the stimulatory effects of diazoxide. We observed a significantly reduced diazoxide-elicited K_ATP_ current in channels containing the WT Lys^1337^ SUR2A subunit (1.28±0.04, *P*<0.05) compared with the WT Ser^1369^ SUR1 subunit (2.10±0.12) in our system (Figures 6A, 6B and 6D). Our data show that the introduction of a positively-charged lysine residue at position 1369 (SUR1 Lys^1369^) significantly reduces the diazoxide-elicited K_ATP_ currents in the absence of ADP (1.41±0.03), a value that is comparable to WT SUR2A Lys^1337^ (1.28±0.04; Figures 6A–6D). Addition of 0.1 mmol/l ADP did not alter the magnitude of diazoxide-induced currents in WT SUR1 Ser^1369^ (Figures 6A and 6D). However, exposure to ADP resulted in a significant increase in diazoxide-induced currents in both the mutant SUR1 Lys^1369^ and WT SUR2A Lys^1337^ containing K_ATP_ channels (Figures 6B–6D). The SUR1 Lys^1369^ single mutation appears to convert the diazoxide pharmacological profile of SUR1 containing K_ATP_ channels towards that of a SUR2A containing K_ATP_ channel phenotype.

**Figure 6:**
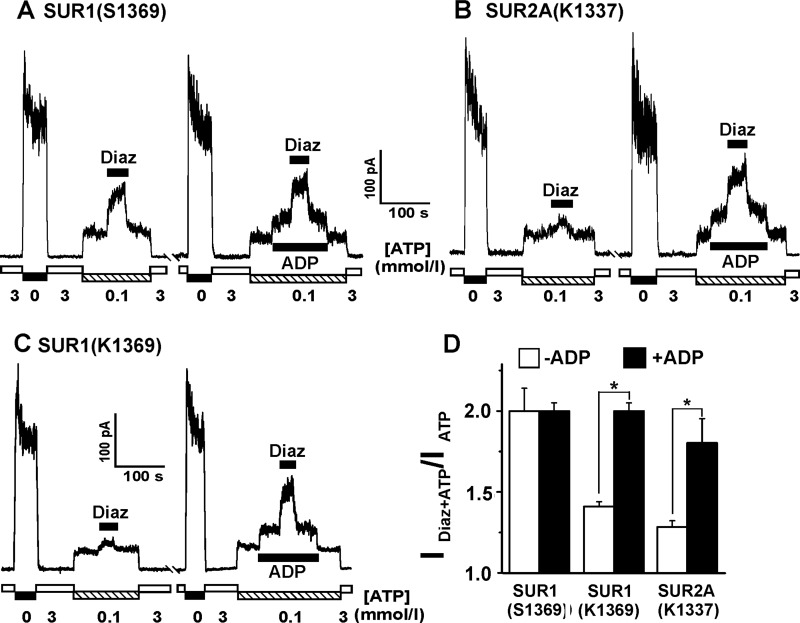
SUR1 amino acid residue at position 1369 determines diazoxide pharmacology of the K_ATP_ channel (**A**) WT SUR1 Ser^1369^ responds in a similar manner in response to 0.1 mmol/l diazoxide in the presence or absence of 0.1 mmol/l ADP. (**B** and **C**) The presence of a positively charged lysine residue in the WT SUR2A Lys^1337^ and the mutant SUR1 Lys^1369^ K_ATP_ channels result in a significant decrease in their response to 0.1 mmol/l diazoxide in the absence of 0.1 mmol/l ADP. (**D**) Grouped data of normalized current (*n*=4–17 patches per group). *P<0.05.

These results also suggest that the reduced effect of diazoxide and overt ADP dependence in the SUR2A-containing K_ATP_ channels may be due to the effects of the hairpin loop Lys^1337^ residue on intrinsic MgATPase activity. Therefore, we investigated GTP-mediated increases in K_ATP_ channel currents and diazoxide sensitivity in WT SUR1 and mutant SUR1 Lys^1369^-containing K_ATP_ channels. Our results show that diazoxide significantly increases GTP-mediated K_ATP_ channel current in the WT SUR1 Ser^1369^ compared with GTP alone (2.83±0.16 compared with 1.86±0.09; *P*<0.01; Figures 7A and 7D). However, introduction of the positively-charged lysine residue at position 1369 (SUR1 Lys^1369^) results in a loss of this diazoxide-elicited increase in GTP-mediated K_ATP_ channel activity (1.36±0.06 compared with 1.23±0.04; Figures 7B and 7D). For comparison, we also performed similar experiments in the SUR1 A1369 T2D susceptibility variant [2] and observed that diazoxide was still able to increase K_ATP_ current further in the presence of GTP (3.19±0.10 compared with 2.87±0.07; *P*<0.05; Figures 7C and 7D). Taken together, these data support the notion that MgATPase activity is required for diazoxide-induced K_ATP_ current activation. To test this concept further, we performed diazoxide experiments in the absence or presence of the MgATPase inhibitor BeF_x_, an ATP γ–phosphate mimetic that is thought to maintain the catalytic region in its pre-hydrolytic conformation [22,23]. As expected, both WT SUR1 and WT SUR2A exhibited a significant decrease in diazoxide-elicited current in the presence of BeF_x_ (1.24±0.02 compared with 2.01±0.08 and 1.05±0.01 compared with 1.27±0.09 respectively; Figures 7E–7G). It should be noted that the overall magnitude of the decrease observed with BeF_x_ is substantially larger in SUR1 compared with SUR2A (Figure 7E–7G), indicating that SUR1 containing K_ATP_ channels possess greater intrinsic MgATPase activity (compared with SUR2A).

**Figure 7:**
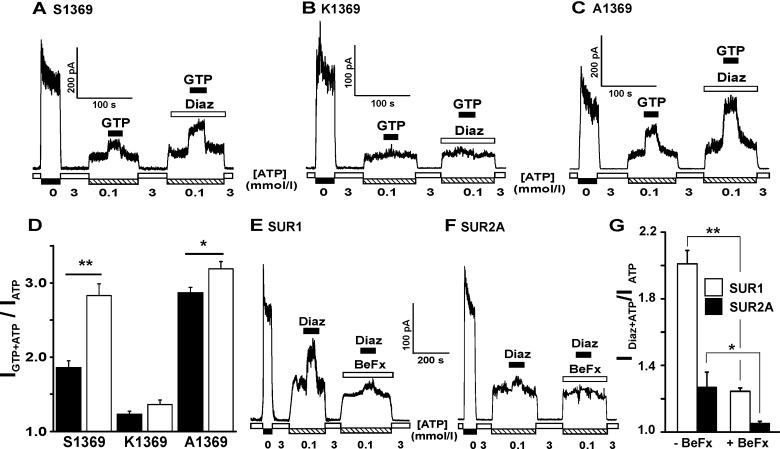
The effect of diazoxide and the MgATPase inhibitor BeFx on GTP-induced K_ATP_ channel function (**A**–**C**) The WT SUR1 Ser^1369^ and Ala^1369^ mutant channels display a significant increase in GTP-induced, MgATPase-dependent channel activation whereas the Lys^1369^ mutant results in a decrease. (**D**) Graphical representation of normalized current. (**E** and **F**) WT SUR1 Ser^1369^ displays much greater inhibition of K_ATP_ channel currents elicited by 0.1 mmol/l diazoxide in the presence of 1 mmo/l of the MgATPase inhibitor BeFx than the WT SUR2A Lys^1337^ channel. (**G**) Graphical representation of normalized current (*n*=5–8 patches per group). *P<0.05 and **P<0.01.

## DISCUSSION

In addition to the regulation of K_ATP_ channel activity by ATP and ADP, the intrinsic enzymatic MgATPase activity, bestowed upon the channel by the SUR subunit, is an important component of the appropriate nt regulation of K_ATP_ channel activity, although the molecular mechanisms that govern MgATPase activity are not fully understood. Molecular insights into the importance of this mechanism can be taken from a previous study on the rare heterozygous SUR1 gene (*ABCC8*) mutations R1380L and R1380C that cause neonatal diabetes via increases in MgATPase activity [9]. *In silico* homology modelling of the catalytic MgATPase regions in SUR1 places Arg^1380^ within the predicted major MgATPase catalytic site in NBD2. These mutations increase MgATPase activity, rendering the K_ATP_ channel markedly less sensitive to MgATP inhibition compared with free ATP inhibition and leading to severely impaired insulin secretion in the afflicted patients [9]. Furthermore, we have previously shown that residue 1369 also regulates MgATPase activity [2], providing a plausible mechanism by which the type 2 diabetes susceptibility variant S1369A in SUR1 may suppress insulin secretion in humans homozygous for the risk variant haplotype E23K/S1369A [24]. The SUR1 NBD1/2 dimer homology model [17] places residue 1369 adjacent to a hairpin loop in the β-sheet that forms the backbone of the NBD2 portion of the major MgATPase catalytic site 2 (Figure 1A). Therefore, substitution of serine with an alanine at residue 1369 (S1369A) may alter the structure and/or flexibility of this region, leading to an increase in MgATPase activity of the K_ATP_ channel. We further speculated that the side chain of Ser^1369^ might form a hydrogen bond with the terminal -NH_2_ group on the side chain of Gln^1372^ (Figure 1A). This hydrogen bond would probably constrain the structure of the hairpin loop and the β-sheet adjacent to the major MgATPase active site in NBD2 (Figure 1A). Substitution of the serine with an alanine at position 1369 (Ala^1369^) removes only the side chain terminal hydroxy group that would remove the ability to hydrogen-bond with Gln^1372^ and reduce a putative structural constraint on the hairpin loop, resulting in the observed increases in MgATPase activity.

Our results (Figures 1–3) provide key evidence that the interaction between the side chains of residues 1369 and 1372 is a key determinant of intrinsic K_ATP_ channel MgATPase activity and that the side chain interaction strength is inversely related to K_ATP_ channel MgATPase activity, with a stronger interaction resulting in weaker enzymatic activity. Cysteine mutagenesis has been used to introduce artificial disulfide bonds that are strong, yet reversible, between closely interacting side chains within ion channels [25,26]. Our results indicate that the reversible formation of a disulfide bond between residues Cys^1369^ and Cys^1372^ that would strengthen side chain interaction results in decreased MgATPase activity (Figure 4).

Interestingly, we observed the greatest reduction in MgATPase activity (measured as reduced channel response to GTP) in K_ATP_ channels containing the SUR1 Lys^1369^ mutation (Figures 3C and 3D). As NBD2 is the major catalytic region of the channel complex, we performed a sequence alignment of NBD2 between all SUR isoforms; SUR1, 2A and 2B (Figure 5A). The only amino acid difference between these isoforms is the presence of a serine at residue 1369 in SUR1, whereas the analogous residue in SUR2A and 2B is a lysine at residue 1337. As there are documented differences in both MgATPase activity and diazoxide sensitivity [3,27,28] we determined whether this single amino acid change from a serine to a lysine is associated with these observed differences using a direct biochemical assay of NBD2 MgATPase activity. The concentration of fully formed catalytically-functional NBD2 dimers could not be determined in these experiments, precluding any analysis of the effects of mutations on *k*_cat_ and thus on *V*_max_. However, as *K*_M_ values are independent of the concentration of functional NBD2 dimers present and since the MgATPase activity meets the requirements of rapid equilibrium behaviour [29], we can use this parameter to glean information on binding affinity of ATP for the MgATPase active site. The observed decrease in *K*_M_ values in NBD2 dimers containing a lysine residue at 1369 (SUR1) or 1337 (SUR2A) suggests that the presence of a positively-charged lysine residue and the increased rigidity of the hairpin loop region increases binding affinity of ATP. In the absence of any change in the catalytic rate constant, this would result in a higher relative MgATPase activity at lower ATP concentrations, as observed previously [30,31]. However, electrophysiological data presented in the present study are consistent with the view that introduction of lysine also reduces *k*_cat_ and thus *V*_max_ for ATP hydrolysis and that the reduction in *k*_cat_ more than offsets any increase in the rate of hydrolysis at sub-saturating concentrations of ATP that results from the reduced *K*_M_ value.

Our results demonstrate that the serine to lysine single amino acid switch at residue 1369 in SUR1 allows the K_ATP_ channel to mimic the diazoxide sensitivity of the WT SUR2A-containing channels that require ADP to be present in order to observe diazoxide stimulation (Figure 6). Furthermore, our results also demonstrate that intrinsic MgATPase activity is required to elicit the full diazoxide stimulatory response (Figure 7.). We speculate that the reduced flexibility resulting from introduction of Lys^1337^ in SUR2A impedes dissociation of substrate from the active site, thereby lowering the first-order substrate dissociation rate constant and thus the *K*_D_ (and *K*_M_) for substrate (Figure 5). Furthermore, it has been suggested previously that the ADP-dependence of diazoxide actions in SUR2A-containing channels is due to the requirement for ADP-binding to lock the NBD2 region in a post-hydrolytic state, rendering channels sensitive to diazoxide's actions [8]. If this is indeed the case, then the requirement for ADP may result from a direct interaction between the positively-charged side chain of Lys^1337^ and the negatively-charged phosphate groups on the ADP moiety. As SUR1-containing channels possess a serine at the analogous NBD2 residue in SUR1 (Ser^1369^, Figure 5A), this putative electrostatic interaction is lost, resulting in higher *K*_M_ values and an ADP-independent diazoxide stimulatory effect (Figures 5–7).

In summary, we demonstrate that it is possible to predict relative MgATPase activities of K_ATP_ channels based on the strength of the side chain interactions between residues 1369 and 1372 in SUR1. These results provide further evidence that flexibility within the hairpin loop region proximal to the major NBD2 catalytic site is an important determinant of intrinsic MgATPase activity, a key regulator of K_ATP_ channel function and provide strong evidence for a plausible molecular mechanism by which the common genetic risk *ABCC8* risk variant Ala^1369^ may lead to increased K_ATP_ channel activity and impaired insulin secretion. Moreover, the different sensitivity to the clinically used drug diazoxide between SUR isoforms seems to be largely determined by a single amino acid in NBD2. Our findings therefore provide novel mechanistic insights into the structural determinants that contribute to K_ATP_ channel activity, common genetic diabetes risk variants and pharmacology.

### Limitations

We used two different assays to measure intrinsic MgATPase activity. The electrophysiological GTP-response assay has the major advantage of allowing us to study fully-assembled hetero-octameric channels in real time. However, this technique provides an indirect measurement of enzymatic activity, as it relies upon the MgATPase catalytic conversion of GTP to GDP, resulting in measurable changes in K_ATP_ channel activity. The direct biochemical assay was used to complement the GTP response assay as it has the advantage of providing a direct measurement of enzymatic activity, albeit in GST-fusion NBD2 dimers only and not in full-length K_ATP_ channel protein subunit complexes (as this would simply not be feasible using a GST-fusion approach for full-length hetero-octameric K_ATP_ channels). The direct biochemical assay is further limited by the fact that comparative *V*_max_ values cannot be obtained in the absence of knowledge regarding the concentration of catalytically functional NBD2 dimers. As we were interested in the effects of residue interactions in fully assembled K_ATP_ channels, we chose to use the GTP response assay for the majority of experiments and selectively employed the biochemical assay to provide further information when required (Figure 5).

## Abbreviations

K_ATP_: ATP-sensitive K^+^
K_ir_: inward rectifying potassium
NBD: nucleotide-binding domain
SUR: sulfonylurea receptor
WT: wild-type
